# Differential Expression of Anthocyanin Biosynthetic Genes and Transcription Factor *PcMYB10* in Pears (*Pyrus communis* L.)

**DOI:** 10.1371/journal.pone.0046070

**Published:** 2012-09-28

**Authors:** Li Li, Zhao-Jun Ban, Xi-Hong Li, Mao-Yu Wu, Ai-Li Wang, Yu-Qian Jiang, Yun-Hong Jiang

**Affiliations:** 1 Key Laboratory of Food Nutrition and Safety (Ministry of Education), Tianjin University of Science and Technology, Tianjin, People's Republic of China; 2 Jinan Fruit Research Institute, All China Federation of Supply and Marketing Cooperatives, Jinan, Shandong, People's Republic of China; 3 College of Forestry and Horticulture, Xinjiang Agricultural University, Urumqi, Xinjiang, People's Republic of China; 4 Institute of Particle Science and Engineering, School of Process, Environmental and Materials Engineering, University of Leeds, Leeds, United Kingdom; Virginia Tech, United States of America

## Abstract

Anthocyanin biosynthesis in various plants is affected by environmental conditions and controlled by the transcription level of the corresponding genes. In pears (*Pyrus communis* cv. ‘Wujiuxiang’), anthocyanin biosynthesis is significantly induced during low temperature storage compared with that at room temperature. We further examined the transcriptional levels of anthocyanin biosynthetic genes in ‘Wujiuxiang’ pears during developmental ripening and temperature-induced storage. The expression of genes that encode flavanone 3-hydroxylase, dihydroflavonol 4-reductase, anthocyanidin synthase, UDP-glucose: flavonoid 3-*O*-glucosyltransferase, and R2R3 MYB transcription factor (*PcMYB10*) was strongly positively correlated with anthocyanin accumulation in ‘Wujiuxiang’ pears in response to both developmental and cold-temperature induction. Hierarchical clustering analysis revealed the expression patterns of the set of target genes, of which *PcMYB10* and most anthocyanin biosynthetic genes were related to the same cluster. The present work may help explore the molecular mechanism that regulates anthocyanin biosynthesis and its response to abiotic stress at the transcriptional level in plants.

## Introduction

Anthocyanins, the main water-soluble pigments in many fruits and vegetables, are a subclass of flavonoids synthesized from hexose through the shikimate, phenylpropanoid, and flavonoid pathways. Anthocyanins impart much of the color and flavor in fruits and vegetables, and also appear to function in protecting against oxidative stress, certain cancers, and other age-related diseases [1]. These presumed health-promoting features could be attributed to the antioxidant properties of these compounds and their chemical structure, which appears ideal for free radical scavenging [2].

Anthocyanin biosynthesis may be affected by light, particularly ultraviolet-B irradiation, and various abiotic stresses such as temperature [3], [4]. Temperature, as an important factor in affecting anthocyanin biosynthesis, has been extensively studied in grape, maize, orange, and apple [5], [6], [7], [8]. In arabidopsis, anthocyanin accumulation is induced by low temperature (LT) [9] and reduced by high temperature [10]. In apples (*Malus domestica*) and pears (*Pyrus communis*), LT increases both the anthocyanin content and the expression of genes related to the anthocyanin biosynthetic pathway [8], [11], [12]. In apples, high temperature prevents the accumulation of cyanidin and UDP-sugars [13], which results in the rapid reduction of anthocyanins, followed by renewed synthesis with cooler temperatures, which causes fluctuations in skin color [11].

Recently, the anthocyanin biosynthetic pathway has been almost completely elucidated and most of the genes involved in anthocyanin biosynthesis and regulation have been isolated and studied in many model plants [14]. These genes are involved in both the early step of dihydroflavonols biosynthesis, such as phenylalanine ammonialyase (PAL), cinnimate 4-hydroxylase (C4H), 4-coumarate: CoA ligase (4CL), chalcone synthase (CHS), chalcone isomerase (CHI), and flavanone 3-hydroxylase (F3H), as well as in the successive reactions in anthocyanin production, such as dihydroflavonol 4-reductase (DFR), anthocyanidin synthase (ANS), and UDP-glucose: flavonoid 3-*O*-glucosyltransferase (UFGT) [15].

Compared with other plants, anthocyanin biosynthesis and accumulation in pears were reported to be more complicated and influenced by fruit cultivar, maturity and environmental factors [3], [16], [17]. Two anthocyanins, cyanidin 3-galactoside and peonidin-3-galactoside, are mainly responsible for the color of pear skin [18]. Five genes that encode these anthocyanin biosynthetic enzymes, namely, PAL, CHS, CHI, F3H, and ANS, as well as UDP-glucose: flavonoid 7-*O*-glucosyltransferase (F7GT), were isolated from the young leaves of ‘Conference’ and ‘Pyrodwarf’ pears [19]. Recently, the *DFR* and *ANS* genes in *Pyrus pyrifolia* have been considered the limiting factors for the skin color of the mildly colored ‘Zaobaimi’ pears [20].

The important role of transcription factors (TFs) in the regulation of the anthocyanin biosynthetic pathway in higher plants has been demonstrated in numerous reports. MYB TFs have been extensively analyzed and shown to play the most important role in regulating anthocyanin biosynthesis. *PyMYB10*, *MdMYB10*, *MrMYB1*, and *IbMYB1* are responsible for color development and appear to be correlated with other anthocyanin-related genes in pears, apples, bayberry and sweet potatoes, respectively [21], [22], [23], [24]. Feng et al. reported a light-induced *PyMYB10* that encodes the R2R3 MYB transcription factor gene in *P. pyrifolia*, which could regulate anthocyanin biosynthesis [3]. In addition, R2R3 MYB TFs have been shown to interact closely with basic helix loop helix (bHLH) TFs, another group of regulatory genes involved in anthocyanin biosynthesis [25]. However, the ubiquitous role of MYB TFs and their transcriptional complex in regulating anthocyanin biosynthesis has yet to be demonstrated.

Although studies into the activation and repression of anthocyanin biosynthesis in pears have shown developmental and environmental control, this has not been characterized at the level of transcriptional regulation to date. To investigate the molecular mechanism that underlies anthocyanin accumulation during pear ripening, the present study focuses on anthocyanin accumulation and the expression of structural genes that encode enzymes located at key points in the anthocyanin biosynthetic pathway, including *PAL*, *CHS*, *CHI*, *F3H*, *DFR*, *ANS*, and *UFGT*, as well as the transcription factor *PcMYB10*, which encodes a putative R2R3 MYB protein. The effect of LT exposure during moderately long storage periods (60 days) on anthocyanin accumulation and gene expression in ‘Wujiuxiang’ pear was also studied with room temperature (RT) exposure as the control.

## Materials and Methods

### Plant materials and treatments

*Pyrus communis* cv. ‘Wujiuxiang’ were collected at four different developmental stages (S1, S2, S3, and S4), i.e., 28, 44, 60 and 76 days after full blooming to fruit harvest during the season from spring through late summer, from 8-year-old trees grown in a local commercial plantation. Freshly harvested pears were washed with distilled water, gently dried with paper towels, and then left to dry at RT for 3 h. Subsequently, the pears harvested at stage S1 were randomly divided into two groups and placed at LT and RT: one group was stored in a ventilated 4°C room with 90% to 95% relative humidity (RH), and the other group placed in a temperature-controlled device at 25°C with 90% to 95% RH. Samplings were carried out at both temperatures before storage (D0) and every 15 d for a total storage period of 60 d (D15, D30, D45, and D60). At each sampling time, five pears were randomly collected from each developmental stage and each storage group, immediately frozen in liquid nitrogen, ground into powder, and immediately stored at −80°C until anthocyanin analysis and RNA extraction.

### Anthocyanin concentration determination

The total anthocyanin concentrations were determined according to the method reported by Goodman et al., with slight modifications [21]. Briefly, frozen ground peel tissue (0.5 g) was homogenized in 0.5 mL of methanol with 0.01% HCl. The resulting homogenate was left overnight in HCl/methanol and then centrifuged for 5 min at 10,000 *g* in a microcentrifuge. Absorbance of the supernatant was measured at 530 nm using a UV-visible spectrophotometer (UV-1600, Shimadzu, Kyoto, Japan).

### RNA extraction and cDNA synthesis

Total RNA was extracted from the ‘Wujiuxiang’ pears using the hot borate method by Wan and Wilkins [23]. The total RNA was quantified spectrophotometrically by measuring the OD 260/280 and OD 260/230, and the integrity of each sample was assessed by electrophoresis on 1% agarose gel containing 0.66 M formaldehyde in the presence of ethidium bromide. To remove genomic DNA contamination, all RNA extracts were treated with DNase I using a DNA-free kit following the manufacturer's instructions (Applied Biosystems, USA). Subsequently, first-strand cDNA was synthesized from the total RNA (2 µg) using oligo (dT) primers using a PrimeScript™ RT-PCR Kit following the manufacturer's instructions (TaKaRa, Japan).

### Real-time quantitative PCR (qPCR) analysis

The differential expression of anthocyanin biosynthetic and regulatory genes (*PcPAL*, *PcCHS*, *PcCHI*, *PcF3H*, *PcDFR*, *PcANS*, *PcUFGT*, and *PcMYB10*) was analyzed using real-time quantitative PCR (qPCR) with an ABI 7500 Real-Time PCR System (Applied Biosystems, USA), according to the manufacturer's recommendations. Each reaction mixture with a final volume of 20 µL was added to each well. An initial hot start was performed at 95°C for 10 min, followed by 40 cycles of 95°C for 30 s, 58°C to 60°C for 1 min, and 72°C for 1 min. All gene-specific primer pairs for the real-time qPCR, which were designed using Primer Express 3.0 (Applied Biosystems, Foster City, CA, USA), are listed in Table 1. Each assay using the gene-specific primers amplified a single product of correct size with high PCR efficiency (90% to 110%) [26]. The individual PCR products were separated on 1% agarose gels and stained with SYBR green I to determine their sizes and ensure that a single PCR product was detected for each primer pair. The actin gene (Accession AF386514) was selected as the reference gene for its consistent transcription level in the fruits.

**Table 1:** Primers used for real-time qPCR analysis in Wujiuxiang pear.

Gene	Accession	Forward (5–3)	Reverse (5–3)
*PcPAL*	DQ230992	CAGAACGGTGCTGTGGAGTC	GTGCTTCAACTTGTGCGTCA
*PcCHS*	DQ901397	CACCCTCAACTTCTACCTTACC	GGGACTTCAACAACCACCAT
*PcCHI*	EF446163	TTCCACCGTCCGTCAAACCT	CATGCCTCCACTGCAACCAC
*PcF3H*	AF497633	AAGGGTGGATTCATCGTGTC	GGTCTTGAAGCAGAAGGGTAAT
*PcDFR*	AY227731	AGCAGGAACTGTGAATGTGG	GTTGGGATAATGGTGATGAAAT
*PcANS*	DQ230994	CCTCAAACTCCTGCTGATTAC	TGACCCACTTTCCTTCATAGA
*PcUFGT*	HM775221	GCTCCATCCTCCGTCGTGTA	AGTCTCCAGCAGCGAGTTCC
*PcMYB10*	EU153575	TCGTCAACAAAGAACGGAAAT	AAGGAACGAACATTTACACCC

All real-time qPCR reactions were normalized using the *C_T_* value that corresponds to the actin gene. The relative expression levels of the target genes were calculated with formula 2^−ΔΔCT^ [27].

### Statistical analysis

Experimental data are the mean ± standard deviation of three replicates of the determination for each sample. The data were subjected to a one-way analysis of variance (ANOVA) using with SPSS version 17.0 (SPSS, Inc.) and Pearson correlation coefficients were calculated. All data were analyzed by a Duncan's multiple-range test at the 0.05 significance level and only significant differences were discussed unless stated otherwise.

### Hierarchical clustering analysis

Gene expression that showed statistical changes related to the developmental stages and storage temperatures during the experimental period were grouped through a two-way hierarchical clustering methodology using the PermutMatrix software [28]. Pearson's distance and Ward's algorithm were used for data aggregation.

## Results

### Anthocyanin accumulation and expression profile of genes during ripening

**Figure 1:**
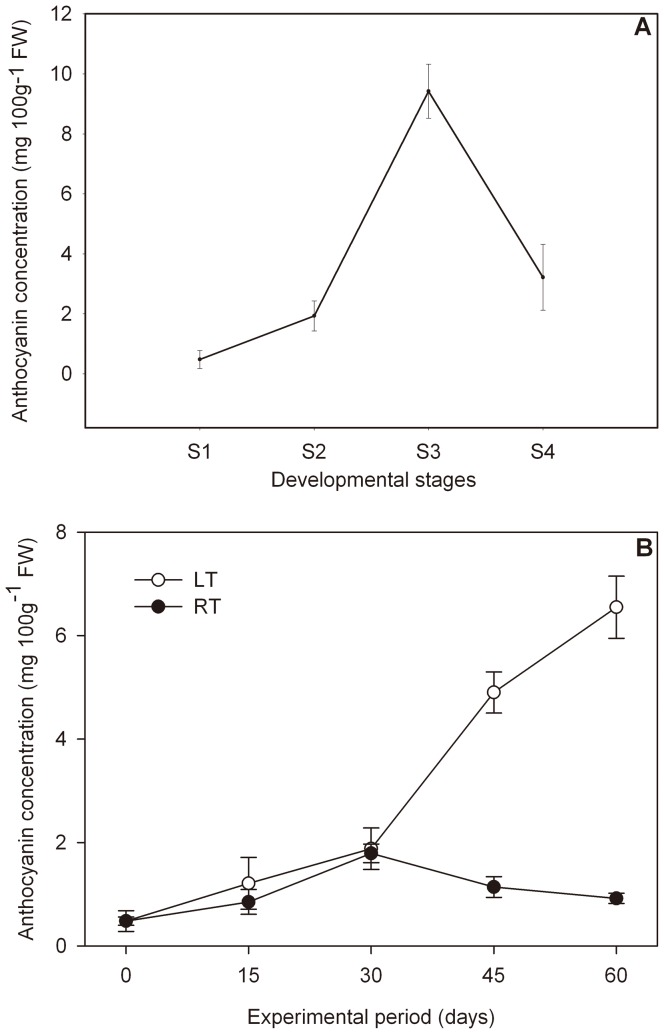
Anthocyanin concentration in ‘Wujiuxiang’ pears. (A): Anthocyanin in pears during ripening; (B): Anthocyanin in pears exposure to LT and RT.

Four developmental stages (from S1 to S4) were investigated to study the pattern of anthocyanin accumulation during pear ripening. The anthocyanin concentration increased greatly with fruit ripening, reaching 9.42 mg 100^−1^ g fresh weight (FW) during the first three stages (S1 to S3), and then dropping during stage 4 (3.22 mg 100^−1^ g FW) (Figure 1A).

**Figure 2:**
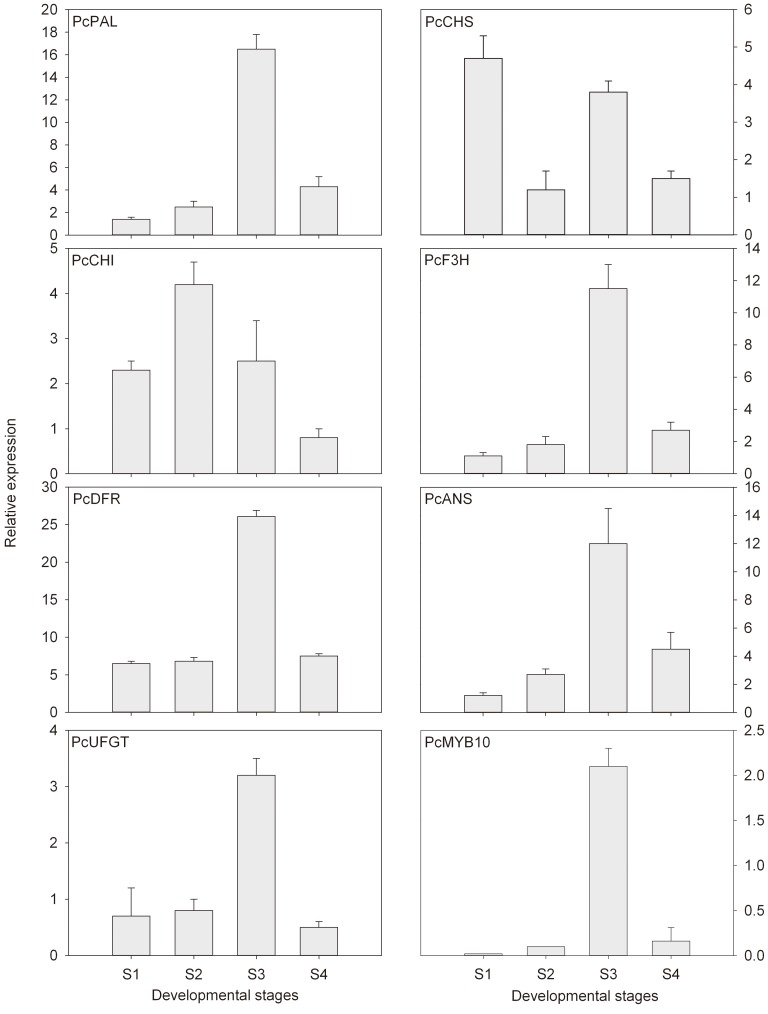
Expression profile of anthocyanin biosynthetic genes during pear ripening. The relative expression levels of target genes were calculated with formula 2^−ΔΔCT^. Each point represents the mean value of three independent experiments performed upon sample triplicates ± SE, with each triplicate derived from mRNA samples independently isolated from five fruits.

**Figure 3:**
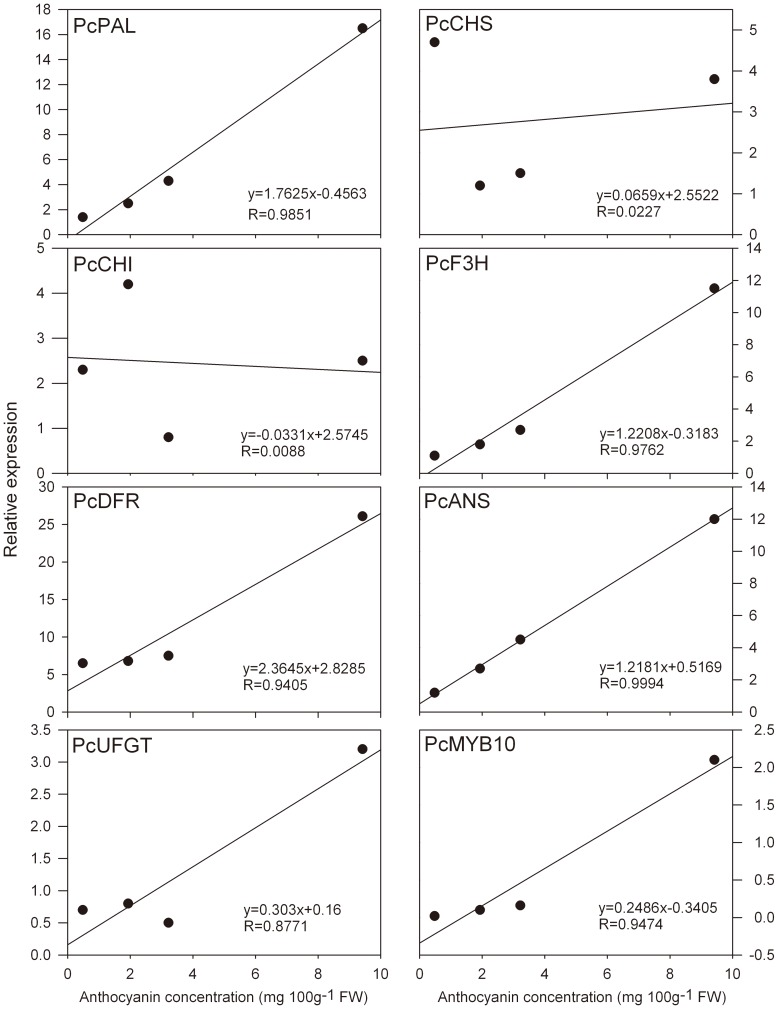
Correlation between anthocyanin concentration and the expression profile of anthocyanin biosynthetic genes in ‘Wujiuxiang’ pears during ripening.

The expression levels of transcripts that encode eight anthocyanin biosynthetic genes (*PcPAL*, *PcCHS*, *PcCHI*, *PcF3H*, *PcDFR*, *PcANS*, *PcUFGT*, and *PcMYB10*) during the developmental stages are shown in Figure 2. In accordance with the pattern of anthocyanin accumulation during ripening, *PcPAL*, *PcANS* and transcription factor *PcMYB10* were expressed at low levels during the first developmental stages, reaching its peak in stage 3, and then declining in stage 4 (Figure 3). In addition, the observed increase in anthocyanin concentration during the developmental stages was accompanied by an increase in the accumulation of *PcF3H*, *PcDFR* and *PcUFGT* transcripts.

### Effect of low temperature (LT) on anthocyanin accumulation and genes expression

The effect of storage temperature on anthocyanin accumulation in pears is described in Figure 1B. We observed that the anthocyanin concentration in pears at RT did not significantly change during the whole experimental period, which showed slight fluctuations between 0.48 and 1.79 mg 100^−1^ g FW. By contrast, the anthocyanin concentration in pears exposed to LT for 60 days sharply increased, reaching a value 12 times higher (6.55 mg 100 g^−1^ FW) that observed on the initial day (Figure 1B), and maintaining values higher than those in samples exposed to RT.

**Figure 4:**
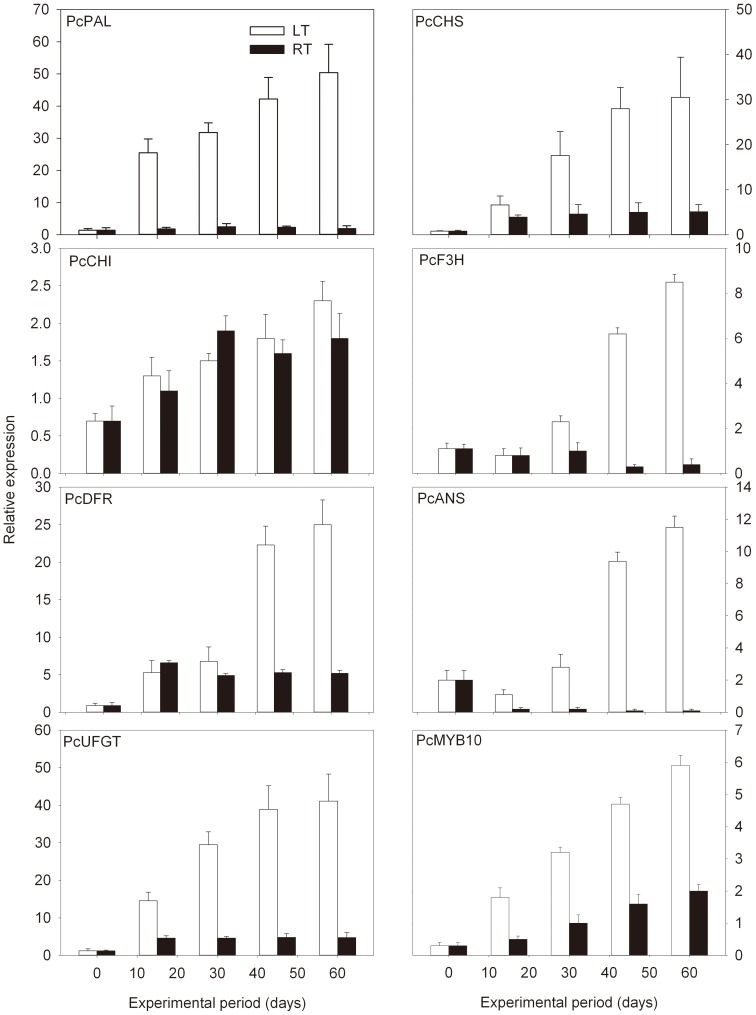
Expression profile of anthocyanin biosynthetic genes in ‘Wujiuxiang’ pears exposed to LT and RT. The sampling and quantitative RT-PCR details are described in Figure 2.

To compare further the differential expression of anthocyanin biosynthetic genes in pears exposed to LT stress, six structural genes and the transcription factor *PcMYB10* were analyzed at LT and at RT. As shown in Figure 4, except for *PcCHI*, the levels of anthocyanin biosynthetic genes *PcPAL*, *PcCHS*, *PcF3H*, *PcDFR*, *PcANS*, and *PcUFGT* were all enhanced under LT conditions compared with those stored at RT. In particular, the expression levels of the upstream gene *PcPAL* and two downstream genes, *PcDFR* and *PcUFGT*, increased by approximately 36-, 27.8-, and 34.3-fold after 60 d storage under LT stress, respectively. However, *PcCHI* expression was not significantly induced by LT. In addition, from the expression profile of the regulatory gene *PcMYB10* in ‘Wujiuxiang’ pears at both LT and RT showed 19.7- and 6.7-fold increases until the end of the experimental period, respectively. Taken together, it is evident that the expression profiles of most structural genes and *PcMYB10* exhibited higher levels at LT than RT. The expression level of *PcMYB10* at LT was almost two times higher than at RT (Figure 4).

**Figure 5:**
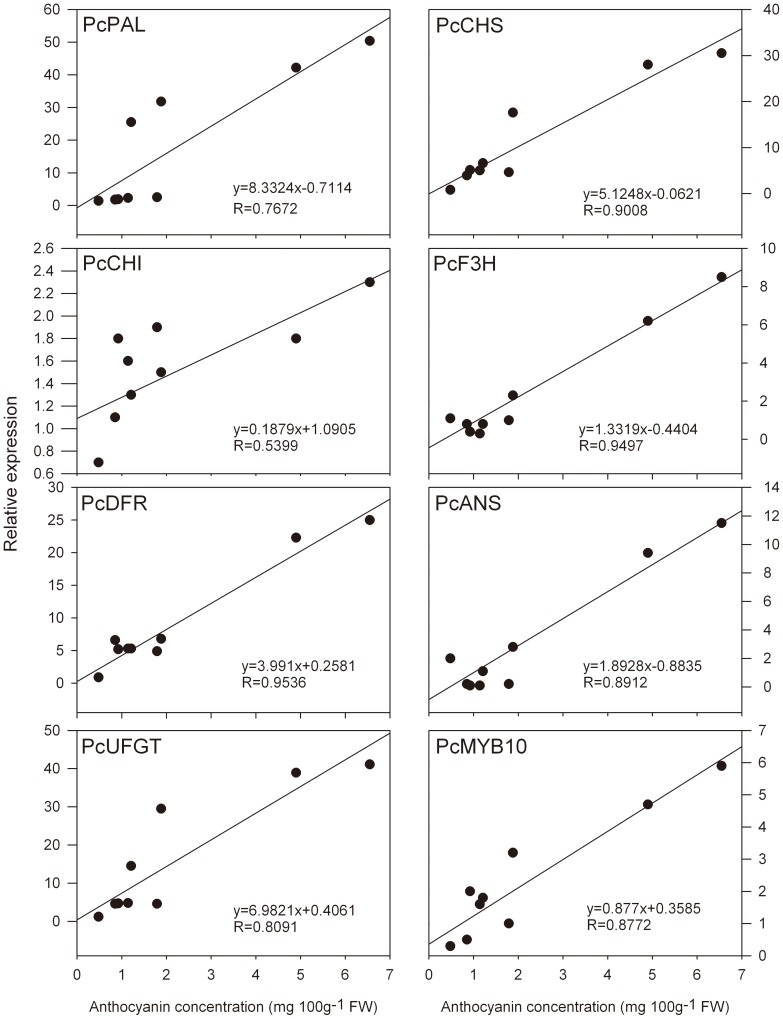
Correlation between anthocyanin concentration and the expression profile of anthocyanin biosynthetic genes in ‘Wujiuxiang’ pears exposed to LT and RT.

To correlate the observed increase in anthocyanin accumulation with the expression of structural and regulatory genes involved in anthocyanin biosynthesis, Pearson's correlation coefficients were calculated in relation to developmental stages and temperature stress (Figures 3 and 5). As shown in both Figure 3 and Figure 5, the expression of the structural genes *PcPAL*, *PcF3H*, *PcDFR*, *PcANS*, and *PcUFGT*, and the regulatory gene *PcMYB10* was positively correlated with anthocyanin concentration (with R values from 0.8091 to 0.9994). The expression of the *PcCHS* gene was strongly positively associated with anthocyanin concentration at LT and RT (R = 0.9008, Figure 5), but not during developmental stages (R = 0.0227, Figure 3). Considering the above, the results suggest that *PcMYB10* is a key gene that controls the anthocyanin biosynthetic pathway and responds to LT stress. Also, it is noteworthy to mention that the expression profiles of structural genes and *PcMYB10* correlated well with anthocyanin concentration at both temperatures (R≥0.5399, Figure 5).

### Hierarchical clustering analysis

**Figure 6:**
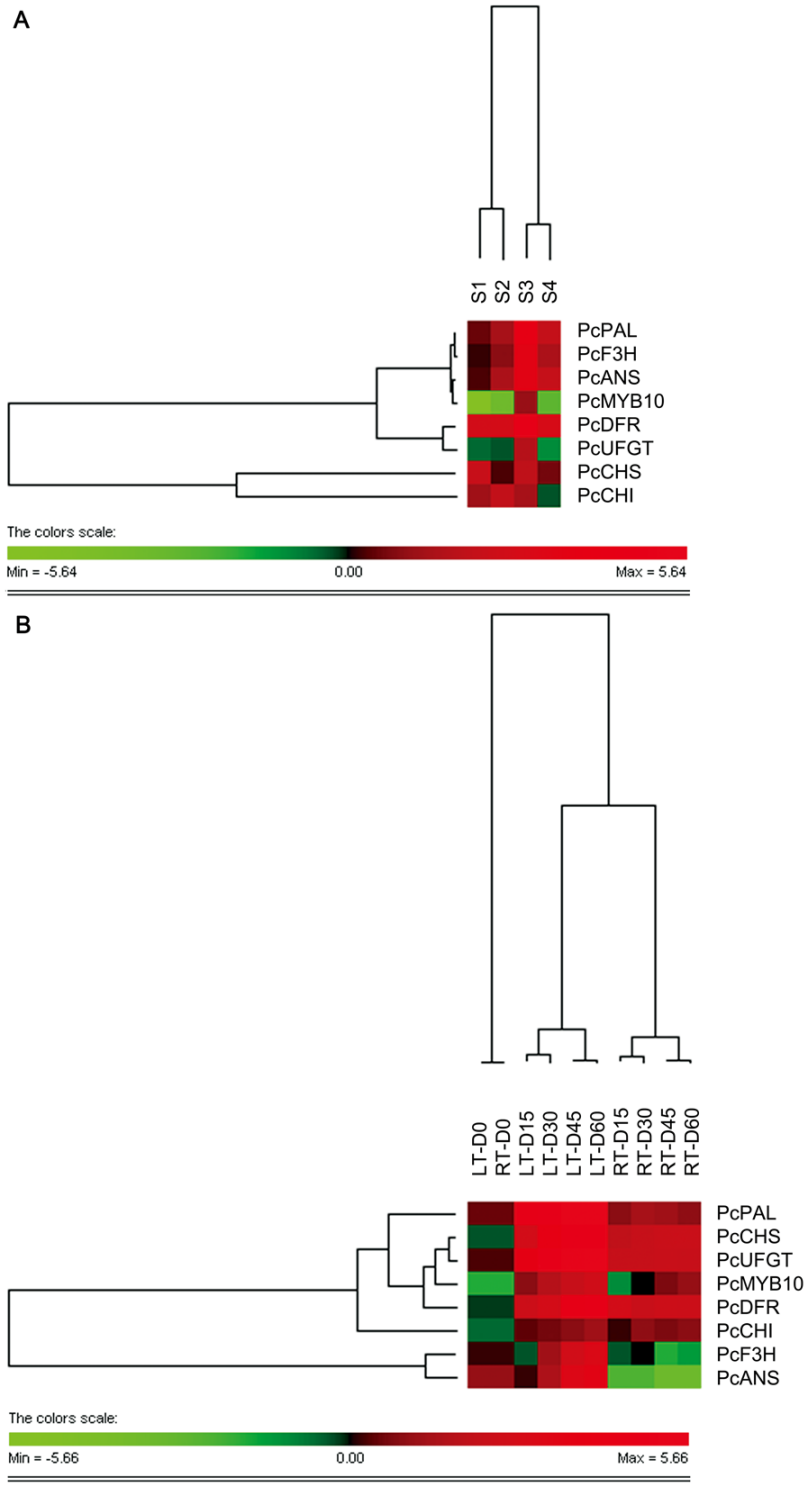
Hierarchical clustering analysis of the log2 of the transcript levels of anthocyanin biosynthetic genes. The clustering analysis was performed on genes differentially expressed both during pear ripening (A) and exposure to LT and RT (B) using PermutMatrix graphical interface, with Pearson's distance and Ward's algorithm for analysis. Red boxes indicate higher levels of expression, and green boxes indicate lower expression levels compared with the Day 0 samples. The color brightness is directly proportional to the expression ratio, according to the color scale at the bottom of the figure.

The differentially expressed genes were subjected to a two-way hierarchical clustering analysis using PermutMatrix software (Figure 6). The clustering of columns, which mirrors the distances among the developmental stages of pear ripening, indicates that the bunch order reflects the sequential succession of samples. However, clear differences were observed between the first two stages (S1 and S2) and the following two (S3 and S4) (Figure 6A). These results suggest that the most significant changes in the expression of anthocyanin-related genes took place between the second (S2) and third stages (S3). For gene expression under both temperatures, the behavior significantly appeared different between LT and RT storage, and was well correlated with the experimental period between 15 and 60 d storage, which indicates evident changes in the expression of anthocyanin biosynthetic genes induced by LT (Figure 6B). As shown in Figure 6A, for row clustering, the cluster describes those that do not show increases during ripening, of which only *PcF3H* and *PcANS* belonged to the same class. In respect to the cold-temperature induction, two different clusters were also observed. One cluster included the transcript factor, *PcMYB10* (Figure 6B).

Taken together from the results of hierarchical clustering analyses, *PcMYB10* is induced by low temperature and shows a significant increase during ripening.

## Discussion

Recently, the available experimental and epidemiologic evidence suggest that anthocyanin has bioactive properties [29]. Previous reports investigated the effects of prolonged refrigerated storage for obtaining therapeutic value-added fruits for human consumption [30]. To explore the molecular mechanism of the anthocyanin biosynthesis response to developmental and environmental control at the transcriptional level, anthocyanin accumulation and the expression profile of anthocyanin biosynthetic genes in ‘Wujiuxiang’ pears were systematically analyzed for the first time in the present study.

Anthocyanin-related genes include the genes that encode the enzymes at the beginning of the general phenylpropanoid biosynthetic pathway (PAL), at the first reaction specifically leading to anthocyanins (CHS), and the consecutive steps (CHI and F3H), in the last branch point that leads to both colored anthocyanins and colorless flavonols (catechins and proanthocyanidins) (DFR), and at the consecutive steps involving anthocyanidin biosynthesis and glycosylation (ANS and UFGT, respectively). In this study, anthocyanin accumulation was positively correlated with the expression of five anthocyanin biosynthetic genes (*PcPAL*, *PcF3H*, *PcDFR*, *PcANS*, and *PcUFGT*) during pear ripening (Figure 3), which indicates that phenolic biosynthetic and multiple downstream genes determine the differential anthocyanin accumulation in ‘Wujiuxiang’ pears. Zhang et al. reported that PAL, CHS, CHI, F3H, DFR, ANS, and UFGT are downregulated in ‘Zaobaimi’ pears but upregulated in ‘Yunhong-1’ pears. CHS may play an important role in the peel coloration of Yunnan red pears (*P. pyrifolia*) [20]. Lister et al. reported that the activities of CHI and UGFT in ‘Splendour’ apples are correlated with anthocyanin accumulation during fruit ripening [31]. However, in ‘Delicious’ and ‘Ralls’ apples exposed to light, UFGT is positively correlated with anthocyanin accumulation, unlike CHS activity [32]. The rapid accumulation of anthocyanin is correlated with an increase in DFR activity in ‘Delicious’ apples [33]. Hence, the different results for the genes involved in fruit anthocyanin biosynthesis may be related to the difference in the genetic background of the materials studied.

Unlike animals and insects, plants do not have their own locomotory system. Plants often synthesize anthocyanin in response to different abiotic stresses, especially in fresh organs, such as fruit [34]. Previous studies reported fast changes in anthocyanin concentration in pears and apples with temperature [8], [35], [36]. In the present study, we confirm the effect of LT stress on anthocyanin accumulation in pear (*P. communis* cv. ‘Wujiuxiang’) and the genes involved in the anthocyanin biosynthetic pathway were expressed to help elucidate the mechanism of anthocyanin biosynthesis response to abiotic stress. The transcript levels of most of the anthocyanin biosynthetic genes remained nearly unchanged in the samples stored at RT, with some cases showing a decreases compared with the day 0 samples. By contrast, in cold-stored samples, the number of transcripts increased sharply after 15 d of storage. After the experimental period, the expression levels ranged between 3.3- and 38.1-fold higher than those in day 0 samples (Figure 4). The correlation between the profile of anthocyanin concentration and the expression of genes involved in their biosynthesis suggests that the increase in gene expression may be mainly, but not uniquely, responsible for the enhancement of anthocyanin accumulation. The pigment peak registered during ripening might also be explained by an increase in the stability of the enzymes rather than an increase in gene transcription.

Extensive studies of *Arabidopsis thaliana*, grapes, apples, pears, petunias, and strawberries show increasing evidence that plants often regulate anthocyanin biosynthesis through the transcriptional control of anthocyanin biosynthetic genes [37], [38]. Anthocyanin biosynthesis in pears are controlled by the MYB transcription factor MYB10 [3]. Very recently, only the R2R3-MYB transcription factors *PyMYB10* and *PcMYB10*, isolated from ‘Aoguan’ pears (*P. pyrifolia*) and ‘Williams’ pear (*P. communis*), respectively, function in anthocyanin biosynthetic regulation [3], [39]. In this study, the transcription of *PcMYB10* in ‘Wujiuxiang’ pears increased during the developmental stages (from S1 to S3) and declined thereafter in stage 4 (Figure 2), in accordance with the change in the pattern of anthocyanin accumulation (Figure 1). This lower efficiency of anthocyanin biosynthesis and the degradation of anthocyanin may explain the fast decline in anthocyanin concentration during the late stage. In addition, both the expression of *PcMYB10* gene and anthocyanin concentration in ‘Wujiuxiang’ pears were induced by LT and maintained at higher levels than exposure to RT, which is closely correlated with anthocyanin accumulation. These results indicate that *PcMYB10* may play an essential role in the transcriptional regulation of anthocyanins and anthocyanin biosynthesis.

In the last two decades, progress has been made in elucidating the coordinated expression pattern of anthocyanin biosynthetic genes controlled at the transcriptional level by MYB and bHLH TFs in other plant species [14], [24], [40], [41], [42], [43]. The *PcMYB10* and bHLH TFs may interact and form transcription complexes that regulate anthocyanin biosynthesis in pears. Our findings point to a direct involvement of the MYB transcription factor *PcMYB10* in pears stored at different temperatures. This result supports the hypothesis that temperature affects anthocyanin biosynthesis in plants via *MYB10*. Multiple anthocyanin regulatory MYB genes have been identified in other plant species, including two alleles of the same gene in apples (*MdMYB10* from ‘Discovery’ [44] and *MdMYBA* from ‘Red Delicious’ [45]) and four closely linked genes in grapes [42].

Overall, based on the evidence above, the transcription factor *PcMYB10* is responsible for developmentally and environmentally controlling anthocyanin biosynthesis in pear fruit. Induction of *PcMYB10* expression is concurrent with the expression of anthocyanin biosynthetic genes during fruit ripening and response to LT stress. To further determine the *PcMYB10* function on the anthocyanin biosynthesis in pears, studies using proteomic and transgenic approaches are necessary.
